# The role of metaboreceptor on exercise in hyperthermic environment with college basketball players

**DOI:** 10.1186/s40064-016-1989-8

**Published:** 2016-03-24

**Authors:** Hyun-Gook Kim, Jong-Kyung Kim, Kyung-Ae Kim, Hosung Nho, Sungchul Lee, Myoung-Jae Chang, Hyun-Min Choi

**Affiliations:** Graduate School of Physical Education, KyungHee University, Seocheon-dong Giheung-gu, Yongin-si, Gyeonggi-do 446-701 Korea

**Keywords:** Metaboreceptor, Total vascular conductance, Temperature environment, Blood pressure, College basketball player

## Abstract

The objective of this study is to review physiological differences of college basketball players cardiovascular responses and group IV metaboreceptor interactions appearing post muscular ischemia exercise (PEMI) caused by a static handgrip exercise (SHE). The subjects were placed in a temperature and moisture stabilized indoor environment for 2 h in order to measure blood pressure. For the SHE, maximal voluntary contraction of arms with a relative strength of 50 % of the maximum muscular strength was put into isometric training for 2 min. After completing the exercises, cuffs worn on the arms of the subjects were pressurized up to 200 mmHg by applying PEMI to block the artery and vein. In this way, the cardiovascular responses created by SHE and PEMI were measured. Blood samples of subjects were collected from the vein of each upper arm before SHE and after PEMI to measure the metabolite hormone and catecholamine in the blood. Results from the measurements showed a significant decrease of blood pressure under high temperature environments compared to normal temperature environments. With respect to PEMI, increases in blood pressure under the high temperature environment were significantly lower compared to the normal temperature environment. In conclusion, this study revealed that college basketball players with good physical strength had higher sensitivities of arterial baroreceptor. However, blood pressure was not increased accordingly because the increase of cutaneous vasoconstriction due to stimuli of the metaboreceptor under a high temperature environment could not be compensated by arterial baroreflex due to the increase of total vascular conductance.

## Background

It is known that the human body uses 20 % of produced energy for the function of the tissue and contraction. The other 80 % of produced energy are converted into heat. To maintain core temperature, the heat generated needs to radiate to the skin exposed to the surrounding environment through blood flow. Heat between the human body and the surrounding environment is constantly transferred to maintain the core temperature. When body temperature increases, cutaneous blood vessels are vasodilated (Wilmore et al. 2008), leading to the relaxation of cutaneous blood vessels. Heat is then emitted from the skin and sweat glands simultaneously to keep our body temperature at its normal level.

In a hyperthermic environment, exercise demands increased blood volume of the skin to radiate heat (Rowell and Blackmon 1986; Rowell et al. 1986), resulting in decreased blood volume returning into the heart. Simultaneously, exercise in a hyperthermic environment increases the cardiac output (CO) to increase blood volume to skeletal muscles, resulting in decreased blood volume into the liver, kidney and skin due to the redistribution of the amount of CO (Crandall 2000). During exercise in a hyperthermia environment, simultaneous physiological responses to increase the blood volume into skin and skeletal muscles. Exercise increases CO and blood volume into liver, internal organs, kidney, and skin. However, continued exercise in a hyperthermia environment increases the blood volume constantly into skin to radiate heat, which decreases CO and exercise performance. The exposure to hyperthermia environment causes syncope due to decreased blood pressure when compared to a normothermic environment because a lot of blood needs to be redistributed into the skin to release heat (Wilmore et al. 2008). Arterial baroreceptor plays an important role in the control of blood pressure. Aerobic exercise training is reported to improves the function of the arterial baroreceptor (Goldberg et al. 2011; Gulli et al. 2003). Although it is known that the arterial baroreceptor function is decreased in a hyperthermic environment (Crandall 2000), not enough research is performed to study the function of arterial baroreflex and the metabolic receptor when well trained athletes are exposed to a hyperthermic environment.

During exercise, sympathetic nerve activity (SNA) increases, followed by the increase of CO, heart rate (HR), blood pressure (BP), and vasoconstriction. These increases are controlled through the following three neural mechanisms: (1) The central command anticipates the exercise and stimulates the cerebral motor cortex, then contracts skeletal muscle and stimulates the activates the cardiovascular control circuits at the same time. The stimulation of this brain stem decreases parasympathetic neural activities, resulting in the increase of SNA, BP, and HR; (2) The increases are regulated by the arterial baroreflex which affects the sympathetic nerve and the parasympathetic nerve at rest or during exercise, which then controls HR, stroke volume (SV), and peripheral blood vessels. This control regulates the blood pressure at every single moment (DiCarlo and Bishop 1992); (3) The increases are controlled by exercise pressor reflex (EPR). EPR is the feedback mechanism delivered from the afferent nerve stimulation of the skeletal muscle group III and group IV receptors during contraction and stretch of the skeletal muscle. Group III mechanoreceptor is stimulated by the muscle contraction and group IV metaboreceptor is stimulated through the metabolite accumulation for exercise. This afferent nerve stimulation increases the blood pressure, HR, CO, and total peripherial resistance (TPR) during exercise (Hayes and Kaufman 2001; Hayes et al. 2005; Stebbins et al. 1988). This study focuses on the neural mechanism of group IV metaboreceptor of the skeletal muscle and the function of arterial baroreflex. It has been reported that the stimulation of the metaboreceptor through static handgrip exercise (SHE) and post exercise muscle ischemia (PEMI) in the normothermic environment or in the hyperthermia environment increases the skin SNA and decreases the cutaneous blood volume (Saito et al. 1990; Vissing and Hjortso 1996; Vissing et al. 1991). Opposite research result is reported that the metaboreceptor stimulation does not affect the cutaneous SNA or contraction of the cutaneous blood vessels (Ray and Wilson 2004). Arterial baroreflex plays a role in the compensation of the increase of blood pressure stimulated by the metaboreceptor. It has been demonstrated that people who have great cardiorespiratory endurance are highly sensitive to arterial pressure (Kim et al. 2005a, b; Raczak et al. 2006).

However, research on exercise and cardiovascular response stimulated by the metaboreceptor using in well trained athletes during a hyperthermic environment is lacking. In addition, arterial baroreceptor in a hyperthermic environment affects muscle metaboreceptor sensitivity is currently unknown. Therefore, this study was designed to examine the difference in physiological response and cardiovascular response at PEMI by SHE in normothermic and hyperthermia environments. In addition, this study determined how arterial baroreceptor would affect muscle metaboreceptor of college basketball athletes in a hyperthermic environment.

## Methods

### Experimental approach to the problem

To obtain reliability of measured data with regard to conditions, data were collected from athletes without any basketball training during off-season. We used the environmental chamber system (The Low Pressure and Hypoxia Center, Yongin, Korea) to set up a goal temperature for the normothermic and hyperthermic environment that might affect blood pressure and body composition since the day before the first visit were controlled. Subjects were exposed to room temperature for up to 2 h to maintain a constant temperature and relative humidity in the normothermic environment setting. After that, body composition, resting blood pressure, and core temperature were measured in subjects as pretest. The core temperature was measured directly through the anus using skin temp and humidity logger (Technox, China). The maximal voluntary contraction (MVC) of the dominant forearm was tested by having subjects squeeze a handgrip dynamometer device (Takei 880, Japan) at maximal effort three times. The highest value was used as MVC. The MVC was used to calculate the relative exercise intensity of 50 % for the experimental protocol. All subjects rested quietly in a chair for at least 5 min and then performed a static handgrip exercise (SHE) by using the principle of isometric training to complete the full 2 min at 50 % of MVC. After SHE, blood pressure cuff induced by inflation was placed around the upper arm to suprasystolic level (~200 mmHg) for application of PEMI (Shibasaki et al. 2001).

The cuff was inflated immediately before the end of SHE for 2 min. Participants’ cardiovascular responses were measured by the method explained above during SHE and PEMI. Examiner collected blood from subjects to measure blood metabolic hormones (lactic acid, La; potassium, K^+^) and catecholamine (epinephrine and norepinephrine) in brachial vein before SHE and after PEMI. 48 h after pretest in the normal temperature environment, all tests were performed in subjects exposed to the hyperthermic environment, including the resting blood pressure, the core temperature, the cardiovascular response during SHE and PEMI, and the responses of blood metabolic hormones and catecholamine.

### Study population

The experiments were performed using 20 healthy college basketball players (age 20 ± 0.2 years). Prior to any intervention, subjects were instructed to abstain from alcohol, caffeine, and strenuous exercise for 24 h. Informed consents were obtained from all subjects before participating. All procedures were reviewed and approved by the Kyung Hee University Institutional Review Board (KHU 2014-19).

### Measurement of body composition variables and core temperature

Subjects were asked to remove any metals, such as jewelry and watch, and stand on the measuring board to determine body composition. Bioelectric impedance method (X-scan Plus II, Korea) was used to measured height, body weight, fat mass, fat free mass, and body fat percentage. In addition, blood pressure was measured by using mercury sphygmomanometer at 10 min after subjects arrived at the laboratory. Examiner measured systolic blood pressure (SBP) and diastolic blood pressure (DBP) twice at 10 min intervals environments (Table 1).

**Table 1:** Physical characteristics of subjects

Variables	Subjects (n = 20)
Age (years)	20 ± 0.2
Height (cm)	189.0 ± 1.9
Body weight (kg)	80.4 ± 1.8
BMI (kg/m^2^)	22.6 ± 0.3
SBP (mmHg)	117 ± 2.7
DBP (mmHg)	63 ± 2.8
MAP (mmHg)	78 ± 3.1
Resting HR (beat/min)	60 ± 1.6
MVC (kg)	49.0 ± 1.4

Values are expressed as mean ± standard error

*BMI* body mass index, *SBP* systolic blood pressure, *DBP* diastolic blood pressure, *MAP* mean arterial pressure, *HR* heart rate, *MVC* maximum voluntary contraction

To measure the core body temperature according to different temperature environments, participants stayed on the environmental chamber system to maintain a constant temperature and relative humidity of the two conditions. The normothermic condition (NC) was 22–23 °C, 50 % of relative humidity (Shibasaki et al. 2001). The hyperthermic condition (HC) was 35 °C, 50 % of relative humidity (Kondo et al. 2000). The core temperatures were measured directly through the anus using skin temp and humidity logger (Technox, China) after subjects have been exposed for up to 2 h in a comfortable condition in each.

### Measurement of cardiovascular responses

Stroke volume (SV), heart rate (HR), SBP, and DBP were continuously measured on a beat-by-beat basis using a Finometer device (Finometer, Finapres Medical Systems, Netherlands). This device uses the Model-flow method to compute a waveform from peripheral arterial pressure to accurately track acute changes in BP and SV via simulation of a non-linear three-element model of vascular input impedance (Bogert and van Lieshout 2005). Arterial BP was measured at the middle finger of the non-dominant hand with the hand positioned at the heart level using servocontrolled finger photoplethysmography. Because finger arterial BP may differ from standard BP measurements, brachial artery BP was also measured by a sphygmomanometer to establish accurate values of resting BP. Mean arterial pressure (MAP) was calculated using the following formula: MAP = [(SBP–DBP) × 1/3] + DBP. CO was calculated from the Fick Equation (CO = SV × HR). Total vascular conductance (TVC) was calculated as CO/MAP. Participants took SHE with previously MVC measuring arm in a comfortable sitting position for 2 min. With previously measured MVC, subjects performed the gripping exercise at 50 % relative intensity of MVC using a dynamometer (Kondo et al. 2003). Participants were instructed to perform the exercise whilst deep breaths. PEMI method has been used to provoke muscle metaboreflex in clinical demonstration (Sausen et al. 2009; Sinoway et al. 1989). To provoke muscle metaboreflex, we blocked brachial vein through increasing the pressure of the cuffs to 200 mmHg by placing the blood pressure cuff on participant’s brachial vein after SHE. The blocking continued for 2 min. Participants were instructed to keep their normal breathing during the blockade of brachial vein and artery.

### Measurement of metabolic hormones and catecholamine

Blood sampling was performed after fasting for at least 8 h (empty stomach). Examiner collected 3 ml of blood from the forearm cardinal vein of each subject using a 22 gauge injection needle. Blood samples were collected from each participant a total of three times using catheter, once per condition (resting, SHE, and PEMI). Blood collecting at the resting condition was carried out when subject visited the laboratory and took a rest for at least 10 min. Examiner also gathered blood using previously inserted catheter immediately after measuring blood pressure at SHE and PEMI. Immediately after collecting the La and K + in EDTA tube, blood samples were degraded to collect plasma by centrifugation at 3000 rpm for 10 min. Collected plasma samples were kept after rapid cooling at −70 °C until ingredient analysis. La was determined by chemiluminescence immunoassay (Cobas Integra-800, Switzerland), and K^+^ by an ion selective electrode assay (Rapodchem 744-Siemens, Germany). Epinephrine and norepinephrine were measured by high performance liquid chromatography (HPLC) method using Acclaim (Bio-Rad, USA) and Plasma catecholamine (Bio-Rad, Germany).

### Statistical analyses

Values of the all items to describe statistics quantity (mean ± standard error) were calculated using Sigma Stat 11.0 program. Cardiovascular responses and plasma concentrations for blood analysis were analyzed using a two-way ANOVA with repeated measures for comparison. If a significant interaction was found, post hoc tests were performed by Tukey’s test. Paired *t* test was used to compare the mean difference regarding the cardiovascular responses and the blood analysis data at rest, SHE, or PEMI in different environments. Statistically significant difference was considered when p-value was less than .05.

## Results

Resting MAP was statistically significant different in the normothermic to the hyperthermic environment (*p* < 0.05). HR and SV were increased slightly but not statistically significant different in both environments. However CO and TVC were statistically significant different in the normothermic to the hyperthermic environment (*p* < 0.05). However, the core temperature at a resting condition in the hyperthermic environment was significantly lower than that in the normothermic environment (Table 2).

**Table 2:** Physiological responses in the normothermic and the hyperthermic environment at resting

Variables	NT	HT
MAP (mmHg)	83 ± 2.9	79 ± 1.6*
HR (beat/min)	64 ± 2.6	65 ± 2.3
SV (ml)	110.2 ± 4.6	117.8 ± 5.9
CO (L/min)	6.6 ± 0.4	7.6 ± 0.4*
TVC (ml/min/mmHg)	80.7 ± 4.7	96.1 ± 5.5*
Core body temperature (°C)	36.5 ± 0.1	37.2 ± 0.1*

Values are expressed as mean ± standard error

*MAP* mean arterial pressure, *HR* heart rate, *SV* stroke volume, *CO* cardiac output, *TVC* total vascular conductance, *NT* normal temperature, *HT* high temperature

* Significantly different versus normal temperature

**Fig. 1:**
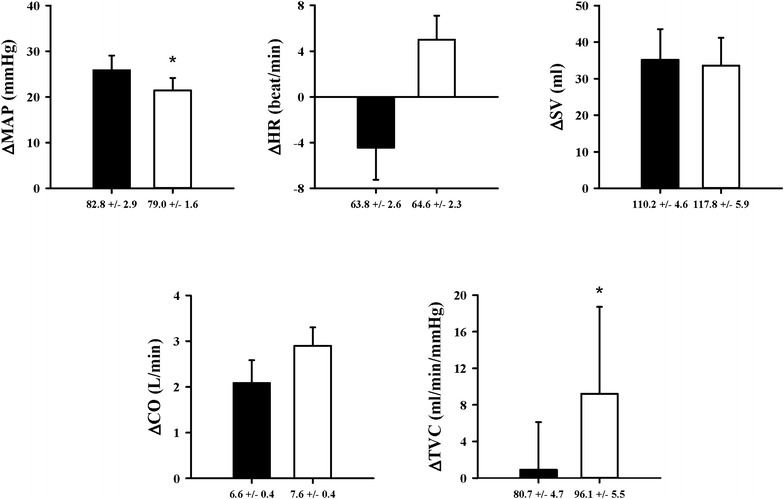
MAP, HR, SV, CO, TVC responses in the normothermic and the hyperthermic environment during SHE; *Black bar* normothermic environment, *Open bar* hyperthermic environment; *significant at *p* < 0.05 compared to the normothermic environment

During SHE as contrasted with the resting, ΔMAP was statistically significant different in the normothermic to the hyperthermic environment (*p* < 0.05). But ΔHR, ΔSV, and ΔCO were not significantly increased in the normothermic to the hyperthermic environment. However ΔTVC were significantly increased in the normothermic to the hyperthermic environment (*p* < 0.05) (Fig. 1).

**Fig. 2:**
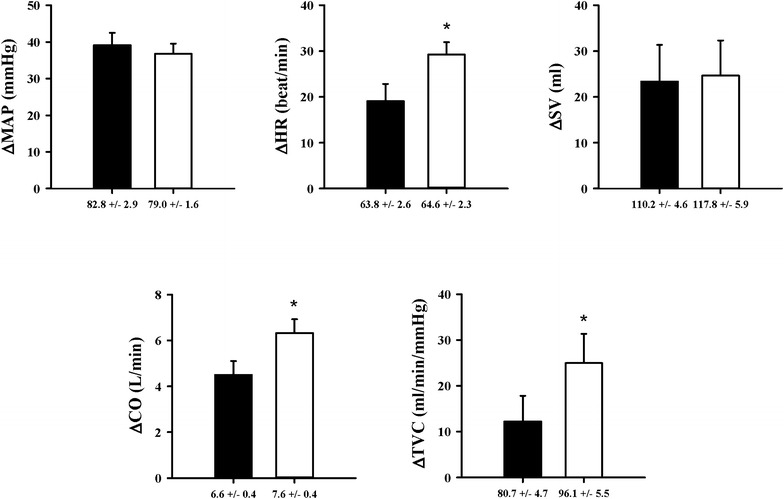
MAP, HR, SV, CO, TVC responses in the normothermic or hyperthermic environment at PEMI; *Black bar* normothermic environment; *Open bar* hyperthermic environment; *significant at *p* < 0.05 compared to the normothermic environment

At PEMI, ΔMAP was not statistically significant different in the hyperthermic to the the normothermic environment. But ΔHR, ΔCO, and ΔTVC were significantly increased in the hyperthermic to the normothermic environment (*p* < 0.05), ΔSV was not significantly decreased in the hyperthermic environment to the normothermic environment (Fig. 2).

**Fig. 3:**
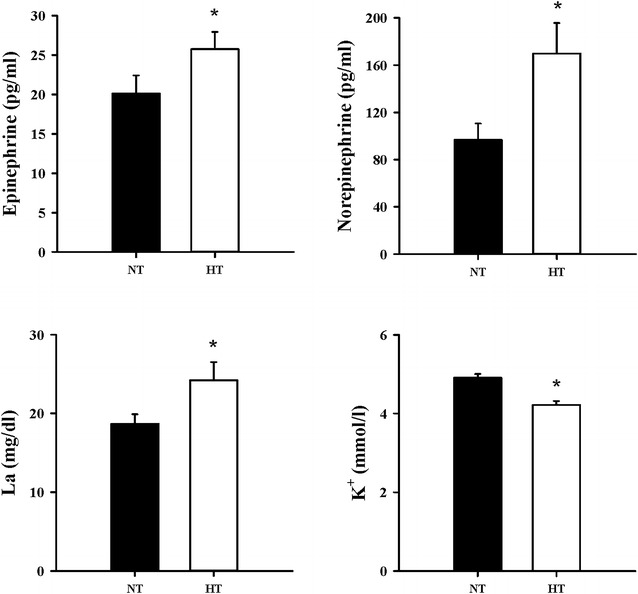
Epinephrine, norepinephrine, La, K^+^ concentrations in the normothermic and hyperthermic environments at resting. *NT* normothermic environment, *HT* hyperthermic environment; *significant at *p* < 0.05 compared to the normothermic environment

Resting levels of norepinephrine and epinephrine were statistically different between normothermic and hyperthermic environment (*p* < 0.05). The concentration of La was significantly increased in the normothermic to the hyperthermic environment. However, the concentration of K^+^ was significantly decreased in the normothermic to the hyperthermic environment (*p* < 0.05) (Fig. 3).

**Fig. 4:**
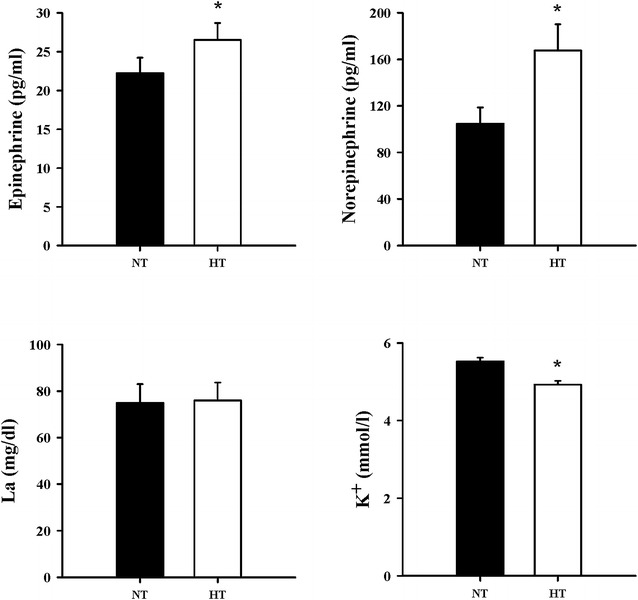
Epinephrine, norepinephrine, La, K^+^ concentrations in the normothermic or hyperthermic environment at PEMI; *NT* normothermic environment, *HT* hyperthermic environment; *significant at *p* < 0.05 compared to the normothermic environment

At PEMI, levels of norepinephrine and epinephrine were statistically different between normothermic and hyperthermic environment (*p* < 0.05). However, the concentration of K^+^ was significantly decreased in the normothermic to the hyperthermic environment (*p* < 0.05) (Fig. 4).

## Discussion

Heat exposure increases blood volume toward cutaneous region in a hyperthermic environment. Blood volume in a hyperthermic environment can be increased to 7500 ml/min from 300 ml/min in a normothmic environment (Rowell and Blackmon 1986). At rest, it is important that CO increases along with TPR increases toward skin and other tissues (i.e. splanchnic, renal) to prevent excessively decreased blood pressure (Rowell and Blackmon 1986; Rowell et al. 1986). Exposure to a hyperthermic environment results in complex response to decreases or keep the blood pressure. Our study showed that MAP was significantly decreased in the hyperthermic environment because the blood vessels toward skin were relaxed. Total peripheral resistance (TPR) was decreased, although CO increased in the cardiovascular response of college basketball players at resting. There is increasing sympathetic activation to non-cutaneous bed to redistribute blood by increasing the blood volume toward skin (Cui et al. 2004; Keller et al. 2006). Generally, CO increment in a hyperthermic environment occurs through HR and SV increase (Damato et al. 1968; Minson et al. 1998; Rowell et al. 1969). Our study revealed that CO increase was due to SV increase. Arterial baroreceptor performs an important function in regulating blood pressure. It has been reported that aerobic exercise training improves the function of the arterial baroreceptor (Goldberg et al. 2011; Gulli et al. 2003). However, the function of the arterial baroreceptor decreases in a hot environment. Although athletes who have higher aerobic fitness have been reported to have improved function of the arterial baroreceptor (Crandall 2000), arterial baroreflex was not able to increase to normal due to the decreasing blood pressure from the TPR decrease after exposure to the hyperthermic environment.

Exercise in the hyperthermic environment increased blood volume to the skin to maintain body temperature. CO was increased to meet this demand. However, there is a limitation of increase in a CO. It is the most challenging issue in the regulation of cardiovascular response to maintain homeostasis in such situation. Abnormal cardiovascular response occurs when untrained persons perform a prolong exercise at a high intensity in a hyperthermic environment (Crandall and González-Alonso 2010). On the other hand, well-trained athletes are adapted to cardiovascular response for exercise in a hyperthermic environment. Usually, the modified cardiovascular response during exercise in a hyperthermic environment occurs in mid to high intensity. Such responses include the decrease of SV, central venous pressure, blood pressure, and CO. However, continuous exercise in a low to moderate intensity maintains or increases CO level (Armstrong et al. 1987; Laughlin and Armstrong 1983). Our study revealed a statistically significant increase in CO and TVC in MAP in the hyperthermic environment compared to the normothermic environment. However, no significant difference in the blood pressure response was found between the two environments. These results suggest that enough CO increase and TPR decrease might have maintained the blood pressure level because participants were well-trained basketball player who performed low-intensity exercises instead of high-intensity in this study. We suggest that the decrease of the blood pressure could occur because TPR decreased to emit heat for exercise in the hyperthermic environment.

In our study, the brachial artery and vein were occluded by a blood cuff immediately after the static exercise to stimulate only metaboreceptors without the effect of the central command and the mechanoreceptors. Metaboreceptor stimulation increases blood pressure with SNA through vascular obstruction. It has been reported that exercise in normal temperature affects blood pressure by CO increase rather than overall TPR increase (Choi et al. 2013; Kim et al. 2005a, b). The stimulation of the skeletal muscle metaboreceptors in the hyperthermic environment does not affect the conduction of nonglabrous cutaneous blood vessels (i.e., hairy skin), whereas the conductance of glabrous cutaneous blood vessels decreases (i.e., nonhairy skin) (Kondo et al. 2003). Blood pressure responses vary depending on the long-term training type. Amano et al. (2011) compared blood pressure responses among male sprinters, male distance runners, and untrained males during PEMI to examine skeletal muscle metaboreceptor in a hyperthermic environment and found that the sprinter group had significantly increased MAP during stimulation of the metaboreceptor compared to the distance runner group or the untrained group. Significant increase of MAP in the sprinter group could be due to the fact that the sprinters have more metabolite accumulation due to their fast developed twitch muscles. In agreement with the results of previous studies, our study also revealed that blood pressure increased through the stimulation of metaboreceptor in the normal temperature and increase of CO. Metaboreceptor stimulation did not affect the conductivity alteration of the cutaneous blood vessels. The cutaneous blood vessels were relaxed, TPR was decreased, and blood pressure was less increased to emit heat. Kim et al. (Kim et al. 2005a, b) stimulated a living dog’s metaboreceptor through the vascular obstruction after removing the arterial baroreflex to examine how the arterial baroreflex and the metaboreflex interacted with each other. They found that increased blood pressure through stimulation of the metaboreceptor almost doubled compared to that before the removing of arterial baroreflex. They also found increase of CO and TPR caused excessive blood pressure increase after the removing of arterial baroreflex. Their study demonstrated that the blood pressure increase by metaboreflex absorbed shock through the arterial baroreflex, which offsets SNA whereby stimulated the peripheral blood vessel. Our study results were in consistent with previous research finding that blood pressure increase by the metaboreflex in a hyperthermic environment is less increased compared to that in a normothermic environment (Crandall 2000). We anticipated that the blood pressure would increase through increased TPR resistance because the function of the arterial baroreflex would be decreased in a hypertehmic environment. However, the opposite result was found. Although this study did not find the clear mechanism, there was possibility that the function of the metaboreceptor was deteriorated because the core temperature increased due to high temperature relaxed the cutaneous blood vessels which increased TVC.

It has been known that the exposure to a hyperthermic environment increases the catecholamine density in the blood or have no effect (Brenner et al. 1997; Powers et al. 1982). Our results revealed that the density of catecholamine was significantly increased at resting. Decreased blood pressure at resting is perceived by arterial baroreflex and the sympathetic nerves (DiCarlo and Bishop 1992). Therefore, the decreased blood pressure in the hyperthermic environment might have increased the sympathetic nerves by the adverse effect of the arterial baroreflex which then increased the concentration of catecholamine. Linnane et al. (2004) and Furtado et al. (2007) considered that different results on this could be due to differences in subjects’ characteristics and core temperatures. The concentration of lactate in the blood is the primary metabolite to arouse the blood vessel relaxation. The present study suggests the possibility that the high concentration of lactate might have caused the decrease in blood pressure at resting through relaxing peripheral blood vessels. A high correlation between MAP increase during the isometric handgrip exercise and the amount of K^+^ released during exercise has been reported (Ooue et al. 2013). However, this study suggests that low K^+^ concentration in the hyperthermic environment might have decreased the blood pressure at rest. Powers et al. (1982) reported that the concentration of catecholamine in the blood and SNA were significantly increased in a hyperthermic environment. This study measured the catecholamine in the blood during PEMI to stimulate the metaboreceptor immediately after exercise and found that the level of catecholamine was significantly increased. Increased SNA was also found after stimulation of the metaboreceptor. It has been reported that the La concentration in the blood during exercise between normal temperature and hyperthermic environment is not significantly different (Backx et al. 2000; Falk et al. 1998). Our study also found no difference in the concentration of lactate in the blood between the two environments. According to a previous research (Daley et al. 2002), MAP increase is highly correlated with the amount of released K^+^ during exercise. Therefore, the decreased blood pressure during PEMI in the hyperthermic environment might have resulted from low K^+^ concentration.

This study derived a conclusion based on results of cardiovascular response by metaboreceptor stimulation during SHE and PEMI in the normothermic and the hyperthermic environments targeting college basketball players. Although the blood pressure significantly decreased at rest in the hyperthermic environment compared to that in the normothermic environment, there was no significant difference in the blood pressure response during SHE in the hyperthermic environment compared to that in the normothermic environment. In addition, the blood pressure increase appeared to be low in the hyperthermic environment than that in the normothermic environment. This study found that the adverse effect of the metaboreceptor was not increased SNA but increased TVC.

## Conclusions

College basketball players who have high physical fitness possess high sensitivity of arterial baroreflex and metaboreceptor stimulation which could increase vasoconstriction toward skin in a hyperthermic environment. The blood pressure was less increased because the arterial baroreflex could not offset peripheral vasodilation.

## Abbreviations

HR: heart rate
SV: stroke volume
CO: cardiac output
TVC: total vascular conductance
TPR: total peripherial resistance
BMI: body mass index
SBP: systolic blood pressure
DBP: diastolic blood pressure
MAP: mean arterial blood pressure
BP: blood pressure
MVC: maximal voluntary contraction
SHE: static handgrip exercise
PEMI: post exercise muscle ischemia
SNA: sympathetic nerve activity
BSA: body surface area
HPLC: high-performance liquid chromatography
La: lactic acid
K^+^: potassium
